# Sex and sexual orientation in relation to tobacco use among young adult college students in the US: a cross-sectional study

**DOI:** 10.1186/s12889-018-6150-x

**Published:** 2018-11-08

**Authors:** Jingjing Li, Regine Haardörfer, Milkie Vu, Michael Windle, Carla J. Berg

**Affiliations:** 1 Department of Behavioral Sciences and Health Education, Rollins School of Public Health, Emory University, 1518 Clifton Road, NE, Room 524, Atlanta, GA 30322 USA; 2 Winship Cancer Institute, Emory University, Atlanta, USA

**Keywords:** Tobacco use, Alternative tobacco product, Sexual orientation, Sex differences, Young adults, College students, Multivariate multiple regression

## Abstract

**Background:**

Sexual minority young adults represent a high-risk population for tobacco use. This study examined cigarette and alternative tobacco product (ATP) use prevalence across sexual orientation (heterosexual, gay/lesbian, and bisexual) among college-attending young adult men and women, respectively.

**Methods:**

Baseline data from a two-year longitudinal study of 3386 young adult college students aged 18–25 in Georgia were analyzed. Correlates examined included sociodemographics (age, sex, sexual orientation, race/ethnicity, college type, and parental education). Outcomes included past 30-day use of tobacco (cigarette, little cigars/cigarillos [LCCs], e-cigarettes, hookah, any tobacco product used, and number of tobacco products used, respectively). Two-group, multivariate multiple regression models were used to examine predictors of tobacco use among men and women, respectively.

**Results:**

Among *men* (*N* = 1207), 34.7% used any tobacco product; 18.6% cigarettes; 12.3% LCCs; 16.8% e-cigarettes; and 14.7% hookah. Controlling for sociodemographics, gay sexual orientation (OR = 1.62, *p* = 0.012) was associated with higher odds of cigarette use; no other significant associations were found between sexual orientation and tobacco use. Among *women* (*N* = 2179), 25.3% used any tobacco product; 10.4% cigarettes; 10.6% LCCs; 7.6% e-cigarettes; and 10.8% hookah. Being bisexual was associated with cigarette (*p* < 0.001), LCC (*p* < 0.001), and e-cigarette use (*p* = 0.006). Lesbian sexual orientation was associated with cigarette (*p* = 0.032) and LCC use (*p* < 0.001). Being bisexual predicted any tobacco product used (*p* = 0.002), as well as number of tobacco products used (*p* = 0.004). Group comparisons showed that the effect of sexual minority status on LCC use was significantly different for men versus women.

**Conclusion:**

Sexual minority women, especially bisexual women, are at higher risk for using specific tobacco products compared to heterosexual women; homosexual men are at increased risk of cigarette use compared to heterosexual men. These nuances in tobacco use should inform interventions targeting sexual minorities.

## Background

Tobacco use is a significant public health problem in the United States. An emerging line of studies suggests that sexual minorities, defined for this study as lesbians, gays, and bisexuals (LGBs), are at higher risk for tobacco use compared to their heterosexual counterparts, especially during young adulthood [1–7]. Despite a relatively robust literature documenting higher risk of tobacco use among sexual minorities, these findings are more complex when considering specific sexual minority groups among the different sexes, as well as the more nuanced alternative tobacco products (ATPs).

First, findings about sexual minorities in the aggregate might not necessarily represent an accurate portrayal of tobacco use profiles among subgroups of this population. Recent work suggests that not all sexual minorities experience the same prevalence and risk of tobacco use [1–4, 8]. In general, evidence across studies shows that bisexuals report a higher tobacco use rate than gay/lesbian and heterosexuals [2, 8, 9]. Secondly, studies stratified by biological sex suggest higher rates of cigarette and ATP use among sexual minority versus heterosexual women, while these differences are not found among men [2, 8, 10]. Additionally, the increasingly prevalent use of ATPs, such as little cigars and cigarillos (LCCs), e-cigarettes, and hookah, further complicates our understanding of populations at high-risk for tobacco use [1–3, 6, 11–14].

Young adulthood is a particularly vulnerable period for sexual minorities, especially college-attending sexual minorities. In transition to college, sexual minority individuals may experience unwelcoming environments and struggle with incivility or harassment [15, 16]. In addition, many sexual minorities might experience challenges in acknowledging, defining, accepting, and disclosing their sexual identity while transitioning to adulthood [17–19]. As multiple stressors increasingly challenge an individual’s coping capabilities, tobacco use may serve as an alternative coping strategy for sexual minority college students who experience “minority stress” [5, 20]. Minority Stress Theory suggests that being a sexual minority leads to excess stress and, consequently, adverse health outcomes including tobacco use [20, 21].

To date, limited research has examined the use of various tobacco products among young adult men and women representing sexual orientation statuses. While examining sexual orientation group disparities in tobacco use, few quantitative analyses have considered the effect of biological sex or included ATPs [11, 22]. Two separate systematic reviews have examined the prevalence and etiology of smoking among sexual minorities thus far [5, 23]; however, neither of them simultaneously examined smoking by sex, sexual orientation, and specific tobacco products.

As a fundamental step toward addressing the gaps in the literature, this study examined the relationship of sexual orientation (distinguishing heterosexual, gay/lesbian, and bisexual sexual orientation) and past 30-day use of tobacco (cigarette, little cigars/cigarillos [LCCs], e-cigarettes, hookah, any tobacco product used, and number of tobacco product used, respectively) among college young adult men and women, respectively.

## Methods

### Participants and procedure

We used baseline data from Project DECOY (Documenting Experiences with Cigarettes and Other Tobacco in Young Adults). The methods employed for sampling and recruitment for Project DECOY have been published elsewhere [24]. Briefly, Project DECOY is a two-year longitudinal cohort study that includes 3418 students (ages 18 to 25) from seven colleges and universities in Georgia. Schools were located in both rural and urban settings and included two public universities/colleges, two private universities, two community/technical colleges, and one historically black university. The registrars’ offices from these campuses provided e-mail addresses for students aged 18 to 25. In three large campuses, 3000 students were randomly selected; in the remaining four campuses, with fewer than 3000 students, the census of students were emailed. The overall response rate was 22.9% (3574/15,607), which ranged from 12.0 to 59.4% at different campuses. Participants were invited to participate in the study via email invitation. This email invitation included detailed information regarding the tasks involved in this study as well as a “confirm” hyperlink. Once participants clicked the “confirm” hyperlink, they were enrolled into the DECOY study. Participants received a $30 gift card for baseline assessment, and they could opt out at any time.

Our project was approved by the Emory University Institutional Review Boards (IRBs) as well as the IRBs of the participating colleges and universities. The current study used the baseline data collected in Fall 2014. From this dataset, we excluded 32 (0.9% of 3418) individuals who self-identified as “other” while answering sexual orientation or gender questions. The current study focused on the remaining 3386 (99.1% of 3418) participants.

### Measures

#### Primary outcomes: Tobacco use

Analyses focused on six past 30-day tobacco use outcomes: cigarette use, LCC use, e-cigarette use, hookah use, any tobacco product use, and number of tobacco products used. Participants were asked: “During the past 30 days, on how many days did you: smoke cigarettes; smoke little cigars or cigarillos; use an e-cigarette; or use a hookah or waterpipe?” Responses were dichotomized into 0 (if days of use for any products = 0) or 1 (if days of use for any products ≥1) for each outcome. Given that the distribution of all ATP outcomes was approximately negative binomial, and only a few participants used ATPs in the past 30 days, we dichotomized them into “non-use” and “any use” in order to model them appropriately. For the cigarette outcome, there were a number of participants who used cigarettes frequently. Therefore, we conducted a sensitivity analysis to assess differences between categorizing cigarette use into three groups (i.e., no-use, used 1–25 days, and more than 25 days) and dichotomizing it (non-use vs. any use). Our results demonstrated that these two categorizations produced only minimal differences in the regression modeling results. For consistency and simplicity, we dichotomized the cigarette use outcome as well. We also constructed two additional outcome variables: 1) any vs. no tobacco product use in the past 30 days; and 2) the number of tobacco products used in the past 30 days (ranging from 0 to 4).

#### Primary predictor: Sexual orientation

Participants were asked in the survey, “Do you consider yourself to be: heterosexual or straight; gay, lesbian, or homosexual; bisexual; or other?” Responses were recoded into dummy variables: heterosexual sexual orientation (1 = yes, 0 = no; reference group), gay/lesbian sexual orientation (1 = yes, 0 = no), and bisexual sexual orientation (1 = yes, 0 = no). Those who selected “other” (*n* = 28, 0.8%) were excluded from the study.

### Stratification variable: Sex

Participants were asked, “What is your gender?” The responses were dichotomized into “1” for men and “2” for women. Participants who selected “other” (*n* = 4, 0.1%) were excluded from this study.

#### Covariates: Sociodemographics

Participants were asked to report their age (continuous variable), race (White, Black, other [including American Indian or Alaskan Native, Asian, Native Hawaiian or Pacific Islander, and those with more than one race]), ethnicity (Hispanic vs. non-Hispanic), the school they attended (coded as public, private, technical, historically black), and highest educational level of either of their parents (coded as bachelor’s degree or greater versus less education).

### Statistical analyses

Univariate analyses were conducted to examine the distribution of each variable. ANOVA and Chi-square tests were used to assess the bivariate relationships between independent variables and six dependent variables (all past 30-day): 1) cigarette use; 2) LCC use; 3) e-cigarette use; 4) hookah use; 5) any tobacco product used; and 6) number of tobacco products used.

To model the use of four types of tobacco products (past 30-day cigarette, LCC, e-cigarette, and hookah use), we used multivariate multiple regression in order to model all four outcomes simultaneously. We used biological sex as the grouping variable; the four types of tobacco use behaviors as dependent variables; sexual orientation as the main predictor; and age, ethnicity, race, school type, and parental education as covariates. In this multivariate multiple regression, no equality constraints were imposed across groups. By modeling four types of tobacco use across men and women simultaneously, we took into account possible differences in variances across groups [25]. We further tested whether men and women differ in the tobacco-sexual orientation associations. Specifically, we compared the parameter estimates of sexual orientation on each tobacco use outcome between men and women by imposing equality constraints across groups using the Wald Chi-square Test [25]. All above analyses were conducted using Mplus 7.4 [25].

To model any tobacco use, we used binary logistic regression; to model the number of tobacco products used, we used Zero-inflated Poisson (ZIP) regression. These analyses were conducted using SAS 9.4. (SAS Institute, Inc., Cary, NC).

## Results

### Participant characteristics

Overall, 3.2% (*n* = 109) identified as gay/lesbian, and 3.8% (*n* = 129) of the respondents identified as bisexual. Among men (35.6%; *n* = 1207), 4.2% reported being gay, and 2.6% reported being bisexual. Among women (64.4%; *n* = 2179), 2.7% reported being lesbian, and 4.5% reported being bisexual.

### Tobacco use among men

Among men, 34.7% reported use of any tobacco products in the past 30 days; 18.6% cigarettes; 12.3% LCCs; 16.8% e-cigarettes; and 14.7% hookah (Table 1). The mean number of tobacco products used was 0.63 (SD = 1.01). Bivariate analyses indicated that gay men had a higher rate of cigarette use than heterosexual men (31.4% vs. 17.8%, *p* = 0.048; Table 2).

**Table 1:** Sociodemographic Characteristics and Past 30-day Tobacco Use in Men (*n* = 1207) and Women (*n* = 2179)

Variables	Total sample M	Men	Women
	(SD) or N (%)	M (SD) or N (%)	M (SD) or N (%)
	*N* = 3386	*N* = 1207	*N* = 2179
Sexual orientation (%)^a^			
Homosexual	109 (3.2)	51 (4.2)	58 (2.7)
Bisexual	129 (3.8)	31 (2.6)	98 (4.5)
Heterosexual	3120 (92.1)	1116 (92.5)	2004 (92.0)
Age (SD)	20.55 (1.97)	20.56 (2.03)	20.54 (1.93)
Race (%)^b^			
Black	827 (24.4)	156 (12.9)	671 (30.8)
White	2110 (62.3)	842 (69.8)	1268 (58.2)
Other	409 (12.1)	195 (16.2)	214 (9.8)
Ethnicity (%)^c^			
Non-Hispanic	3110 (91.8)	1113 (92.2)	1997 (91.6)
Hispanic	252 (7.4)	88 (7.3)	164 (7.5)
School type (%)			
Public university	924 (27.3)	411 (34.1)	513 (23.5)
Private college/university	1309 (38.7)	574 (47.6)	735 (33.7)
HBCU	410 (12.1)	49 (4.1)	361 (16.6)
Technical college	743 (21.9)	173 (14.3)	570 (26.2)
Parental education (%)^d^			
<Bachelor’s degree	1625 (48.0)	438 (36.3)	1187 (54.5)
≥Bachelor’s degree	1717 (50.7)	753 (62.4)	964 (44.2)
Cigarettes (%)	450 (13.3)	224 (18.6)	226 (10.4)
vs. No	2936 (86.7)	983 (81.4)	1953 (89.6)
LCC (%)	379 (11.2)	149 (12.3)	230 (10.6)
vs. No	3007 (88.8)	1058 (87.7)	1949 (89.4)
E-cigarettes (%)	368 (10.9)	203 (16.8)	165 (7.6)
vs. No	3018 (89.1)	1004 (83.2)	2014 (92.4)
Hookah (%)	413 (12.2)	177 (14.7)	236 (10.8)
vs. No	2973 (87.8)	1030 (85.3)	1943 (89.2)
Any tobacco product used (%)	970 (28.7)	419 (34.7)	551 (25.3)
vs. No	2416 (71.3)	788 (65.3)	1628 (74.7)
Number of tobacco products used (SD)	0.48 (0.87)	0.62 (1.01)	0.39 (0.78)

*Participants indicated “Don’t know” and “refused to answer”: ^a^ 0.8 (*n* = 28); ^b^ 1.2% (*n* = 40); ^c^ 0.7% (*n* = 24); ^d^ 1.3% (*n* = 44)

**Table 2:** Bivariate Analyses Examining Differences Between Participants Identifying as Heterosexual vs. Homosexual vs. Bisexual

Variables	MEN				WOMEN			
	Heterosexual *N* = 1116	Homosexual *N* = 51	Bisexual *N* = 31	*p*	Heterosexual *N* = 2004	Homosexual *N* = 58	Bisexual *N* = 98	*p*
Age (SD) ^a^	20.56 (2.05)	20.80 (1.88)	20.03 (1.6)	.245	20.54 (1.92)	20.50 (2.06)	20.49 (2.00)	.953
Race (%) ^b^								
Black	137 (12.4)	12 (23.5)	6 (19.4)	.107	602 (30.4)	27 (47.4)	37 (37.8)	.022
White	785 (71.2)	34 (66.7)	19 (61.3)		1175 (59.3)	26 (45.6)	56 (57.1)	
Other	181 (16.4)	5 (9.8)	6 (19.4)		204 (10.3)	4 (7.0)	5 (5.1)	
Ethnicity (%) ^b^								
Non-Hispanic	1035 (93.1)	46 (90.2)	25 (83.3)	.100	1835 (92.4)	54 (93.1)	91 (93.8)	.852
Hispanic	77 (6.9)	5 (9.8)	5 (16.7)		152 (7.6)	4 (6.9)	6 (6.2)	
School type (%) ^b^								
Public university	382 (34.2)	15 (29.4)	11 (35.5)	.031	473 (23.6)	15 (25.9)	20 (20.4)	.223
Private college/university	535 (47.9)	20 (39.2)	15 (48.4)		689 (34.4)	14 (24.1)	28 (28.6)	
HBCU	40 (3.6)	6 (11.8)	3 (9.7)		327 (16.3)	15 (25.9)	18 (18.4)	
Technical college	159 (14.3)	10 (19.6)	2 (6.4)		515 (25.7)	14 (24.1)	32 (32.7)	
Parental education (%) ^b^								
< Bachelor’s degree	397 (36.1)	24 (47.1)	12 (38.7)	.275	1086 (54.9)	35 (61.4)	57 (59.4)	.436
≥ Bachelor’s degree	703 (63.9)	27 (52.9)	19 (61.3)		894 (45.1)	22 (38.6)	39 (40.6)	
Cigarettes (%) ^b^	199 (17.8)	16 (31.4)	5 (16.1)	.048	192 (9.6)	11 (19.0)	22 (22.5)	<.001
vs. No	917 (82.2)	35 (68.6)	26 (83.9)		1812 (90.4)	47 (81.0)	76 (77.5)	
LCCs (%) ^b^	140 (12.5)	6 (11.8)	2 (6.5)	.591	185 (9.2)	19 (32.8)	24 (24.5)	<.001
vs. No	976 (87.5)	45 (88.2)	29 (93.5)		1819 (90.8)	39 (67.2)	74 (75.5)	
E-cigarettes (%) ^b^	189 (16.9)	9 (17.7)	4 (12.9)	.830	146 (7.29)	3 (5.2)	15 (15.3)	.011
vs. No	927 (83.1)	42 (82.3)	27 (87.1)		1858 (92.7)	55 (94.8)	83 (84.7)	
Hookah (%) ^b^	164 (14.7)	7 (13.7)	4 (12.9)	.946	211 (10.5)	7 (12.1)	18 (18.4)	.050
vs. No	952 (85.3)	44 (86.3)	27 (87.1)		1793 (89.5)	51 (87.9)	80 (81.6)	
Any tobacco used (%) ^b^	386 (34.6)	21 (41.2)	8 (25.8)	.362	477 (23.8)	23 (39.7))	48 (49.0)	<.001
vs. No	730 (65.4)	30 (58.8)	23 (74.2)		1527 (76.2)	35 (60.3)	50 (51.0)	
Number of tobacco products used (SD) ^a^	0.62 (1.01)	0.74 (1.06)	0.48 (0.96)	.509	0.37 (0.75)	0.70 (0.96)	0.81 (0.97)	<.001

^a^Bivariate analysis was ANOVA

^b^Bivariate analysis was Chi-square

In multivariable regression controlling for age, race, ethnicity, school type, and parental education (Table 3, upper panel), gay sexual orientation was significantly associated with higher odds of cigarette use among men (OR = 1.62, 95% CI: 1.11–2.37). Being Black was associated with lower odds of LCC (OR = 0.71, 95% CI: 0.51–0.99) and e-cigarette use (OR = 0.58, 95% CI: 0.40–0.84). Sexual orientation did not predict “any” vs. “no tobacco product used” among men, nor did it predict number of tobacco products used (Table 4, upper panel).

**Table 3:** Multivariate Multiple Regression Analyses Predicting Tobacco Use Behavior in the Past 30 Days among Men (*N* = 1207) and Women (*N* = 2179)

Variables	Cigarettes				LCCs				E-cigarettes				Hookah			
	OR	95% CI		*p*	OR	95% CI		*p*	OR	95% CI		*p*	OR	95% CI		*p*
MEN																
Sexual orientation (ref = heterosexual)																
Homosexual	1.62	1.11	2.37	.012	0.89	0.52	1.50	.650	1.05	0.69	1.59	.825	0.96	0.61	1.49	.847
Bisexual	1.02	0.60	1.75	.932	0.69	0.34	1.42	.316	0.85	0.47	1.52	.573	0.88	0.49	1.59	.677
Age	1.00	0.96	1.05	.960	0.94	0.89	0.99	.030	0.93	0.89	0.97	.001	0.98	0.94	1.03	.528
Hispanic	0.89	0.63	1.26	.516	0.69	0.44	1.08	.105	1.26	0.91	1.75	.160	1.23	0.87	1.72	.235
Race (ref = White)																
Black	0.71	0.51	0.99	.041	0.93	0.66	1.32	.686	0.58	0.40	0.84	.004	1.17	0.86	1.60	.323
Other	1.11	0.88	1.40	.370	0.93	0.71	1.24	.630	0.90	0.71	1.15	.415	1.29	1.01	1.66	.042
School type (ref = private)																
Public	0.99	0.81	1.20	.881	1.23	0.98	1.53	.067	1.05	0.86	1.29	.611	1.23	1.00	1.53	.056
HBCU	0.96	0.51	1.81	.900	2.27	1.34	3.85	.002	1.75	1.00	3.08	.050	1.38	0.83	2.30	.210
Technical	1.63	1.24	2.14	<.001	1.31	0.94	1.82	.106	1.76	1.33	2.31	<.001	1.02	0.74	1.40	.929
Parental education (ref = ≥ Bachelor’s)																
< Bachelor’s	1.15	0.94	1.40	.166	1.01	0.81	1.26	.931	1.16	0.96	1.41	.134	1.13	0.91	1.39	.267
WOMEN																
Sexual orientation (ref = heterosexual)																
Homosexual	1.61	1.04	2.49	.032	2.22	1.55	3.17	<.001	0.89	0.50	1.58	.697	1.00	0.63	1.60	.993
Bisexual	1.84	1.38	2.46	<.001	1.85	1.32	2.59	<.001	1.57	1.13	2.17	.006	1.31	0.94	1.82	.111
Age	1.05	1.01	1.09	.017	0.97	0.92	1.01	.118	0.99	0.95	1.04	.682	0.99	0.95	1.03	.638
Hispanic	0.94	0.68	1.28	.685	0.72	0.47	1.09	.121	0.99	0.71	1.39	.960	1.15	0.87	1.52	.330
Race (ref = White)																
Black	0.54	0.43	0.69	<.001	1.69	1.37	2.08	<.001	0.63	0.48	0.81	<.001	1.40	1.14	1.73	.002
Other	0.77	0.57	1.04	.088	1.21	0.85	1.71	.286	0.88	0.63	1.23	.447	1.39	1.07	1.79	.013
School type (ref = private)																
Public	1.13	0.91	1.41	.253	1.49	1.17	1.92	.002	1.36	1.07	1.72	.011	1.45	1.18	1.78	<.001
HBCU	1.17	0.83	1.66	.374	1.82	1.35	2.44	<.001	1.46	1.01	2.11	.044	1.40	1.07	1.84	.016
Technical	1.74	1.42	2.14	<.001	1.45	1.13	1.88	.004	1.64	1.29	2.09	<.001	1.18	0.95	1.47	.132
Parental education (ref = ≥ Bachelor’s)																
< Bachelor’s	0.86	0.72	1.02	.082	0.83	0.69	1.00	.046	0.88	0.73	1.06	.183	1.01	0.85	1.19	.948

**Table 4:** Binary Logistic Regression Analyses Examining Any Tobacco Product Used in the Past 30 Days and Zero-inflated Regression Analyses Predicting the Number of Tobacco Products Used in the Past 30 Days Among Men (N = 1207) and Women (N = 2179)

Variables	Any Tobacco Product Used				Number of Tobacco Products Used							
					(Count Portion)				(Zero-inflated Portion)			
	OR	95% CI		*p*	OR	95% CI		*p*	Beta	95% CI		*p*
MEN												
Sexual orientation (ref = heterosexual)												
Homosexual	1.29	0.72	2.30	.120	1.06	0.66	1.70	.825	0.77	0.31	1.96	.588
Bisexual	0.67	0.29	1.52	.228	1.12	0.55	2.30	.754	1.76	0.59	5.26	.310
Age	0.95	0.90	1.01	.130	0.95	0.90	1.01	.105	1.02	0.92	1.12	.769
Hispanic	1.11	0.69	1.80	.666	0.93	0.61	1.43	.756	0.80	0.35	1.82	.588
Race (ref = White)												
Black	0.72	0.46	1.12	.077	0.93	0.62	1.40	.737	1.47	0.77	2.83	.243
Other	1.17	0.84	1.65	.096	0.97	0.72	1.31	.856	0.79	0.46	1.37	.404
School type (ref = private)												
Public	1.21	0.91	1.61	.103	1.05	0.83	1.33	.697	0.82	0.53	1.26	.360
HBCU	2.54	1.23	5.25	.045	1.10	0.55	2.20	.796	0.31	0.07	1.39	.125
Technical	1.63	1.09	2.44	.569	1.52	1.09	2.11	.013	1.80	0.45	1.41	.435
Parental education (ref = ≥ Bachelor’s)												
< Bachelor’s	0.87	0.66	1.15	.326	0.85	0.67	1.09	.194	1.02	0.66	1.56	.946
WOMEN												
Sexual orientation (ref = heterosexual)												
Homosexual	1.92	1.10	3.35	.757	1.41	0.90	2.23	.136	0.63	0.24	1.61	.334
Bisexual	3.05	1.99	4.67	.002	1.31	0.96	1.80	.093	0.32	0.15	0.70	.004
Age	1.01	0.96	1.07	.701	0.98	0.92	1.04	.438	0.96	0.87	1.06	.419
Hispanic	0.95	0.62	1.46	.815	0.87	0.53	1.42	.573	0.89	0.40	1.95	.766
Race (ref = White)												
Black	1.25	0.94	1.66	.423	0.59	0.40	0.88	.009	0.36	0.15	0.88	.025
Other	1.22	0.83	1.77	.669	1.29	0.80	2.08	.292	0.62	0.32	1.22	.168
School type (ref = private)												
Public	2.03	1.51	2.73	.333	1.15	0.82	1.62	.407	0.51	0.32	0.83	.006
HBCU	2.50	1.68	3.72	.016	1.29	0.80	2.08	.292	0.46	0.17	1.23	.122
Technical	2.33	1.71	3.17	.012	1.12	0.81	1.56	.486	0.37	0.22	0.63	<.001
Parental education (ref = ≥ Bachelor’s)												
< Bachelor’s	1.11	0.88	1.39	.391	1.26	1.00	1.58	.050	1.10	0.75	1.61	.624

### Tobacco use among women

Among women, 25.3% used any tobacco product in the past 30 days; 10.4% cigarettes; 10.6% LCCs; 7.6% e-cigarettes; and 10.8% hookah (Table 1). The mean number of tobacco products used was 0.39 (SD = 0.78).

In bivariate analyses (Table 2), compared to heterosexual women, both lesbians and bisexual women reported higher use rates of cigarettes (19 and 22.5% vs. 9.6%, *p* < 0.001), LCCs (32.8 and 24.5% vs. 9.2%, *p* < 0.001), hookah (12.1 and 18.4% vs. 10.5%, *p* = 0.05). Bisexual women also reported higher e-cigarette use rate compared to heterosexual women and lesbians (15.3% vs. 7.29% vs. 5.2%, *p* = 0.011). Additionally, bisexual women compared to lesbian and heterosexual women were more likely to report use of any tobacco products (49.0% vs. 39.7 and 23.8%, respectively) and also reported higher mean number of tobacco products used.

Among women, regression results (Table 3, lower panel) indicated that lesbian sexual orientation is associated with higher odds of using cigarettes (OR = 1.61; 95% CI: 1.04–2.49) and LCCs (OR = 2.22, 95% CI: 1.55–3.17). Compared to heterosexual women, women identifying as bisexual were more likely to use cigarettes (OR = 1.84; 95% CI: 1.38–2.46), LCCs (OR = 1.85, 95% CI: 1.32–2.59), and e-cigarettes (OR = 1.57, 95% CI: 1.13–2.17). Black women were less likely to use cigarettes (OR = 0.54, 95% CI: 0.43–0.69) and e-cigarettes (OR = 0.63, 95% CI: 0.48–0.81). However, Black women reported higher odds of using LCCs (OR = 1.69, 95% CI: 1.37–2.08) and hookah (OR = 1.40, 95% CI: 1.14–1.73). Being bisexual predicted any tobacco product used (OR = 3.05, CI: 1.99–4.67, *p* = 0.002; Table 4, lower panel), as well as number of tobacco products used (ZIP Beta = 0.32, CI: 0.15–0.70, *p* = 0.004).

### Two-group (men vs. women) comparison results

We also tested whether men and women differ in the tobacco-sexual orientation associations. The sexual orientation parameter estimates among the sample of men and women were compared using a two-group comparison technique using the Wald Chi-square Test. The two-group (men vs. women) parameter comparison results suggest that biological sex magnifies the association of sexual minority status with negative tobacco use outcomes (Table 5). Specifically, being a woman magnifies the effect of lesbian (*p* = 0.005) and bisexual (*p* = 0.015) identities in increasing LCC use. In terms of other types of tobacco use outcomes, no significant between-sex differences were found.

**Table 5:** Comparison of Parameter Estimates Between Different Sexes (Men vs. Women) Using Wald Test

Variables	Tobacco Use Outcomes											
	Cigarettes			LCCs			E-cigarettes			Hookah		
	Value	df	*p*	Value	df	*p*	Value	df	*p*	Value	df	*p*
Sexual orientation (ref = heterosexual)												
Homosexual	0.001	1	.979	7.995	1	.005	0.198	1	.656	0.019	1	.889
Bisexual	3.589	1	.058	5.915	1	.015	3.285	1	.070	1.307	1	.253
Age	2.496	1	.114	0.499	1	.480	3.983	1	.046	0.036	1	.850
Hispanic	0.045	1	.833	0.015	1	.902	1.028	1	.311	0.087	1	.769
Race (ref = White)												
Black	1.644	1	.200	8.277	1	.004	0.121	1	.728	0.893	1	.345
Other	3.609	1	.058	1.282	1	.258	0.015	1	.901	0.141	1	.707
School type (ref = private)												
Public	0.883	1	.347	1.329	1	.249	2.535	1	.111	1.135	1	.287
HBCU	0.288	1	.592	0.515	1	.473	0.283	1	.595	0.002	1	.964
Technical	0.151	1	.697	0.247	1	.619	0.130	1	.719	0.593	1	.441
Parental education (ref = ≥ Bachelor’s)												
< Bachelor’s degree	4.779	1	.029	1.806	1	.179	4.006	1	.045	0.696	1	.404

## Discussion

This study examined the association between sexual orientation and use of various tobacco products, with the goal of advancing the literature regarding how biological sex and sexual minority status (gay/lesbian sexual orientation and bisexual sexual orientation) are associated with cigarette and ATP use. Our results indicated that some sexual minority subgroups are at a higher risk for using specific tobacco products. However, sexual minority women subgroups reported greater tobacco use differences compared to sexual minority men.

Among young adult men, gay sexual orientation, but not bisexual orientation, was significantly correlated with cigarette use. This finding is consistent with prior research indicating gay men were significantly more likely to smoke cigarettes, while bisexual men were no different from heterosexual men [23, 26]. However, neither gay nor bisexual orientation was associated with LCC, e-cigarette, or hookah use. Evidence has been inconsistent for ATP use among sexual minority men. For example, Emory et al. (2015) reported bisexual men were more likely to use LCCs, while gay men were less likely to use them compared to heterosexual men [2]. In contrast, Johnson and colleagues (2016) found no statistically significant difference between sexual minority men and straight men in cigar use. Instead, they found sexual minority men reported a higher rate of ever using e-cigarettes and hookah than heterosexual men [1]. The differences between findings in these aforementioned studies and current findings are difficult to discern. However, it is possible that, in the current study, the smaller number of men in the sexual minority subgroups and the small number of ATP users among men limited our statistical power to detect potential associations between sexual orientation and ATP use.

In young adult women, lesbian sexual orientation was associated with cigarette and LCC use; bisexual sexual orientation was associated with cigarette, LCCs, and e-cigarette use. These findings align with existing literature that sexual minority statuses among women are associated with the use of a range of tobacco products [1–3, 6, 8]. This includes data drawn from a nationally representative sample of 17,087 U.S. adults, which showed that lesbians were more likely to use regular cigars and LCCs compared to heterosexual women, and bisexual women were more likely to use e-cigarettes and LCCs [2]. Our results also echo previous findings [2, 6, 8] that bisexual women might be experiencing the greatest tobacco use disparities compared to other sexual minority groups or heterosexual men and women. From the perspective of Minority Stress Theory, it is plausible that bisexual women may experience unique stressors (e.g., exclusion from heterosexual and lesbian communities) that may lead to tobacco use [27].

Race was consistently found to be a significant correlate of multiple tobacco use outcomes among young adults, especially among women in our study. However, the sample size limitation restricted further exploration attempts on how race might interact with sexual orientation as well as biological sex in influencing tobacco use. Similarly, Blosnich et al. (2011) reported a higher rate of LCCs and hookah use among Black sexual minorities compared to white sexual minorities [28]. Previous meta-analyses also suggest race is a statistically significant correlate of overall substance use among sexual minorities but do not specify tobacco use [29]. However, more evidence is needed to understand the interaction of multiple identities (i.e., sexual orientation, biological sex, race, etc.) and tobacco use disparities. School type was also a significant correlate of tobacco use in our study. Among young adult men and women attending technical college, a two-year institution, the likelihood of using cigarettes and e-cigarettes in the past 30 days was significantly higher than four-year institutions. One explanation could be that technical colleges typically represent a group of students with lower socioeconomic status (SES) compared with four-year education institutions [30] and lower SES is associated with an escalated use of tobacco [31].

Furthermore, results from the two-group comparison analysis suggest that the sexual orientation – LCC use association is statistically different between men and women, indicating being a woman magnifies the influence that being a sexual minority has on LCC use. However, for use of cigarettes, e-cigarettes, and hookah, the effect of sexual minority status did not differ between men and women. A few studies on tobacco use have documented such between-sex heterogeneity among sexual minorities. For example, Bandiera (2013) suggested that lesbian/gay and bisexual sexual orientation correlated with cigarette use among women but not among men [6]. However, none compared this heterogeneity statistically [1–3, 6, 8–10]. Our findings suggest that between-sex differences might be mostly prominent in LCCs use rather than other types of tobacco use in sexual minority young adults. One study suggested that LCCs are more appealing to young adults for the variety of available sweet and fruit flavored LCCs are more palatable, and using LCCs can enhance mood [32]. Bisexual women reported higher levels of depressive symptoms than gays and lesbians did, and lesbians reported higher levels of depressive symptoms than gays did [33, 34]. Based on Minority Stress Theory, it is possible that sexual minority women, especially bisexual women, use LCCs more often to deal with symptoms of depression or stress more broadly.

Nonetheless, the causes for the between-sex difference across sexual minority groups remain largely unknown. Matthews et al. (2014) found neither childhood victimization, depression, nor anxiety explained differences in smoking prevalence among sexual minority women [35]. One possible explanation may be exposure to tobacco marketing [36–38]. A 2018 study found that sexual minorities more frequently reported exposure to tobacco-related advertisements and anti-tobacco messages [39]. Dilley et al. (2008) suggested that sexual minority women were exposed to more tobacco marketing and also were more receptive to tobacco industry marketing than heterosexual women while sexual minority men were more exposed but no more receptive than heterosexual men [40]. Similarly, Fallin et al. (2015) also found higher tobacco advertising receptivity among sexual minority women, especially bisexual women [41]. It is, therefore, possible that tobacco marketing may target not only sexual minority as a whole, but also specific sexual minority subgroups [2]. Efforts to understand the drivers of tobacco use disparities among sexual minority women, especially bisexual women, are warranted.

The current results indicating tobacco product heterogeneity across sexual minorities emphasize the importance of understanding how newer forms of tobacco products may differentially appeal to these subpopulations. Although ATPs have become increasingly popular in the U.S. [13, 14, 42, 43], the majority of existing studies on tobacco use and sexual minority often do not examine the types of tobacco products or just simply assess participants’ cigarette smoking status [4, 7, 8, 23, 35, 44, 45], making it difficult to assess the actual tobacco-related risks among disadvantaged populations such as sexual minorities. Moreover, while the specific health risks of ATPs warrant additional research, it is important to note that no tobacco is safe to use and that the newer forms of tobacco products (i.e., ATPs) do have distinctive health risks (i.e. nicotine dependence potential and toxicity) [46]. Future research should consider ATPs in addition to cigarette use to address tobacco use disparities in sexual minorities accurately.

The current study findings add to the relatively sparse literature by providing evidence on the distinctive profiles in sexual minority use of tobacco products, as well as pinpointing most at-risk subgroups for tobacco use. Despite experiencing higher rates of tobacco use, public tobacco cessation services were underused by sexual minorities [47]. Additionally, tobacco interventions that directly target sexual minorities are scarce [22, 48–51]. Recent systematic reviews suggest that sexual minority tailored tobacco interventions, including communication campaigns and individual and group counseling programs, appear to be effective [22, 52]. However, qualitative data suggest that sexual minority communities have specific needs regarding tobacco cessation, such that programs designed to facilitate quitting should be tailored to their needs [53]. More importantly, interventions are favored when customized for their specific community (e.g., lesbian or bisexual women) rather than sexual minorities as a whole or the general population [52]. Thus, culturally-competent and inclusive messaging tailoring certain sexual minority subgroups and their patterns of tobacco use may be more effective in recruiting these subgroups. In particular, since we found that bisexual women may be more prone to tobacco use, especially LCC use, interventions might target reductions in LCC use to address the tobacco use disparities among bisexual women.

Although this study has important findings, some limitations should be noted. Although this sample was comprised of diverse young adults in terms of race, ethnicity, and socioeconomic backgrounds; diverse college campus types (e.g., private, public, two-year, HBCU); and students in rural and urban settings, the sample was comprised of college students from Georgia, limiting the generalizability of our results to the broader young adult population. However, it is also important to note that estimated state prevalence of current tobacco use among Georgian young adults (18–24 years old) and Georgian adults in general are not significantly different from national estimates [54, 55]. We also had a relatively low overall response rate (22.9%), partly because we met our sampling quota target in short time intervals (ranging from 1 day at the private schools to 7 days at the technical colleges). As such, our sample may not be representative of the entire college student population in Georgia. Our sample is largely reflective of the student populations of each school, with one exception: our study enrolled fewer male student participants (36%) than female participants (64%), which was disproportionate relative to the student enrollment at the colleges included in the study. However, given that tobacco use is less prevalent among women, having a larger proportion of women allowed us to examine tobacco use with more power. Additionally, the parent study was not designed to address this research aim and thus did not aim to specifically target recruitment of sexual minority populations or assess relevant factors related to tobacco use among sexual minorities, limiting our ability to comprehensively examine the underlying factors for sexual minority tobacco disparities. Finally, the cross-sectional design of this study can only suggest associations rather than provide tests of causality. In terms of causality, no known theoretical basis has suggested sexual orientation itself causes or conveys risks for tobacco use. Instead, it is most likely that the social environment contributes to the tobacco use disparities among sexual minorities [20, 56–58]. To conclude, further research with larger samples of diverse young adults is needed to more comprehensively examine profiles of tobacco use, as well as the underlying mechanisms of use over time.

## Conclusions

Among a sample of young adult college students, results indicated that among men, gay sexual orientation, but not bisexual sexual orientation, was significantly associated with cigarette use. Among women, lesbian sexual orientation was associated with cigarette and LCC use; bisexual sexual orientation was associated with cigarette, LCC, and e-cigarette use. Furthermore, the sexual orientation – LCC use association is statistically different between men and women, indicating being a woman magnifies the influence of being a sexual minority on LCC use.

Our findings provided evidence on the distinctive tobacco use patterns between heterosexual, gay/lesbian, and bisexual young adult college students, and documented that bisexual women might be experiencing the greatest tobacco use disparities compared to other young adult sexual minority groups or heterosexuals. Interventions might target reductions in LCC use to address differences in tobacco use rates among sexual minority young adult women. Further studies are needed to understand the cause for differences in tobacco use prevalence among sexual minorities.

## Abbreviations

ATP: alternative tobacco product
CI: Confidence Interval
DECOY: **D**ocumenting **E**xperiences with **C**igarettes and **O**ther Tobacco in **Y**oung Adults
E-cigarettes: electronic cigarettes
HBCU: Historically Black Colleges/Universities
IRB: Institutional Review Board
LCC: little cigars/cigarillos
LGBs: lesbians, gays, and bisexuals
OR: Odd Ratio
